# Metabolomic profiling of wild rooibos (*Aspalathus linearis*) ecotypes and their antioxidant-derived phytopharmaceutical potential

**DOI:** 10.1007/s11306-024-02103-4

**Published:** 2024-04-14

**Authors:** C. Wilkinson, J. Brooks, M. A. Stander, R. Malgas, R. Roodt-Wilding, N. P. Makunga

**Affiliations:** 1 Department of Botany and Zoology, Stellenbosch University, Private Bag X1, Matieland, 7600 South Africa; 2 Department of Biochemistry, and Mass Spectrometry Unit, Central Analytical Facility, Stellenbosch University, Private Bag X1, Matieland, 7600 South Africa; 3 Department of Conservation Ecology and Entomology, Stellenbosch University, Private Bag X1, Matieland, 7600 South Africa; 4 Department of Genetics, Stellenbosch University, Private Bag X1, Matieland, 7600 South Africa

**Keywords:** Ethnobotany, Phenolics, Metabolomics, Ecotypes

## Abstract

**Introduction:**

*Aspalathus linearis* (commonly known as rooibos) is endemic to the Cape Floristic Region of South Africa and is a popular herbal drink and skin phytotherapeutic ingredient, with health benefits derived primarily from its unique phenolic content. Several, seemingly habitat-specific ecotypes from the Cederberg (Western Cape) and Northern Cape have morphological, ecological, genetic and biochemical differences.

**Objectives and methods:**

Despite the commercial popularity of the cultivated variety, the uncultivated ecotypes are largely understudied. To address gaps in knowledge about the biochemical constituency, ultra-performance liquid chromatography-mass spectrometry analysis of fifteen populations was performed, enabling high-throughput metabolomic fingerprinting of 50% (v/v) methanolic extracts. Antioxidant screening of selected populations was performed via three assays and antimicrobial activity on two microbial species was assessed. The metabolomic results were corroborated with total phenolic and flavonoid screening of the extracts.

**Results and discussion:**

Site-specific chemical lineages of rooibos ecotypes were confirmed via multivariate data analyses. Important features identified via PLS-DA disclosed higher relative abundances of certain tentative metabolites (e.g., rutin, aspalathin and apiin) present in the Dobbelaarskop, Blomfontein, Welbedacht and Eselbank sites, in comparison to other locations. Several unknown novel metabolites (e.g., *m/z* 155.0369, 231.0513, 443.1197, 695.2883) are responsible for metabolomic separation of the populations, four of which showed higher amounts of key metabolites and were thus selected for bioactivity analysis. The Welbedacht and Eselbank site 2 populations consistently displayed higher antioxidant activities, with 2,2-azino-bis (3-ethylbenzothiazoline-6-sulphonic acid) (ABTS) radical scavenging activities of 679.894 ± 3.427 µmol Trolox/g dry matter and 635.066 ± 5.140 µmol Trolox/g dry matter, respectively, in correlation with a high number of phenolic and flavonoid compounds. The contribution of the individual metabolites to the pharmacological effectiveness of rooibos remains unknown and as such, further structural elucidation and phytopharmacological testing is thus urgently needed.

**Supplementary Information:**

The online version contains supplementary material available at 10.1007/s11306-024-02103-4.

## Introduction

*Aspalathus linearis,* popularly known as rooibos, (Burm. F.) R. Dahlgren is a Fynbos plant of the Fabaceae family, endemic to the Cape Floristic Region and one of only a few South African medicinal plants to have been cultivated on a commercial scale for use in the development of phytopharmaceutical products (Van Wyk, 2011). The health benefits of rooibos are derived from its phytochemical properties, namely, the low tannin content, high antioxidant activity, high flavonoid content and the absence of caffeine (Govender, 2007; Joubert & de Beer, 2011). Knowledge of rooibos and its use originated with the descendants of the Khoi and San indigenous people groups, by whom landscapes of the Cape Floristic Region were populated during pre-colonial times. Rooibos has traditional value in South Africa as a medicinal beverage that is used to treat stomach ailments, eczema and other skin conditions, and for relief of colic in infants (Malongane et al., 2018). Various other health benefits from the consumption of rooibos have been reported in literature, namely its anti-diabetic, anti-cancer, anti-aging and more recently anxiolytic properties (Erickson, 2003; Joubert et al., 2008; van Wyk, 2011; Lall & Kishore, 2014).

Rooibos as an economic, ecological and biocultural asset may be linked to the health benefits, and therefore, metabolomic characteristics of the plant. A clearer understanding of the metabolomic differentiation that exists in wild populations of rooibos also serves the aim of conserving those ecotypes that have unique chemical characteristics, as they may contain qualitative and quantitative differences that hold unique bioactivity. Apart from this, the complexity of rooibos extracts alludes to the potential for discovery of new chemical entities that may have pharmacological activity. There has thus been a considerable effort to better define the chemical profile of rooibos that is commercially cultivated, and collections of rooibos that have been consumed and exported as tea for more than a century (Masike et al., 2022; Stander et al., 2017, 2019). A study by Jolley et al. (2017) described a sensory wheel of rooibos, alluding to 17 aroma attributes detected over several years and cultivation regions. This provides evidence of the vast degree of variety that exists within the species. Although both the Northern Cape and Western Cape regions were studied for rooibos cultivation, the sensory profiles were not easily delineated and distinguishable based on the production area.

Currently used industry chemical markers for rooibos product quality control include aspalathin and nothofagin, the former of which is unique to rooibos. Phenylpropenoic acid glucoside (PPAG) is also unique to rooibos, however, it is currently not used as a chemical marker in the monitoring of commercial rooibos products. In recent years, metabolite profiling of *A. linearis* has been exploited by researchers for its potential as a new standard for quality control purposes in routine testing. Metabolomics-based studies on the chemical diversity of wild rooibos ecotypes as explored by Stander et al. (2017) in the illustration of the distinct secondary metabolite profiles of 17 *A. linearis* populations from the Clanwilliam and Wupperthal regions in the Western Cape, and Nieuwoudtville in the Northern Cape, appears equally interesting. Although previous studies on rooibos chemistry have been performed (Beelders et al., 2012; Rabe et al., 1994; Stander et al., 2017; van Heerden et al., 2003), there are still populations of rooibos that are yet to be studied, as these plants often occur in geographically isolated locations of limited accessibility (Hawkins et al., 2011).

Wild rooibos populations are particularly vulnerable to climate change and landscape conversions for agricultural land-use (Patrickson et al., 2008). These populations may be better adapted to changing climate conditions due to their phenotypically plastic nature and a comparative analysis that comprehensively assesses metabolite profiling of these plants from disparate environments may provide some insights into climate-adaptation strategies that are associated with wild populations (Lötter & le Maitre, 2014).

Rural communities living in close proximity to these wild populations are predicted to be more at risk in terms of their livelihood as a result of the decline in these distinct ecotypes. Two communities especially productive in the industry are, for example, the Heiveld Co-operative in the Suid-Bokkeveld near Nieuwoudtville, and the longer established Wupperthal Original Rooibos Tea Co-operative in the Cederberg. Loss of genetic diversity and the possibility of inbred populations, highlighted by Potts (2017) for the commercially valuable sister genus, *Cyclopia* (honeybush species), would weaken future possibilities for land-users to sustain their livelihoods (Lötter & le Maitre, 2014). A number of ecotypes of *A. linearis* have been identified. These differ in respect to their fire survival strategies (i.e., reseeding or resprouting types), morphology, ecology, and their underlying genetic and chemical makeup (Brooks et al., 2021; Hawkins et al., 2011; Malgas et al., 2010; Stander et al., 2017). Certain ecotypes, such as the Dobbelaarskop populations near Nieuwoudtville, have previously been confirmed to have notable genetic differences as compared to the other ecotypes tested (Brooks et al., 2021). The chemical differences in the ecotypes have not yet been characterised sufficiently, despite the recent work on this subject by Stander et al. (2017). Differing quantities in metabolites in tested ecotypes in that particular study were shown but there still remains several ecotypes which have not yet been the subject of any phytochemical studies, therefore providing additional validation for this project.

Several authors have used morphometrics or morphotypes as a basis to differentiate rooibos ecotypes. Links have been made between phytochemistry and product characteristics (e.g.: the colour and the “strength” of the tea) followed by the connection between local rooibos names and morphometrics and how this relates to ecological knowledge of the ecotypes (van der Bank et al., 1995; van der Bank et al., 1999; van Heerden et al., 2003; Malgas et al., 2010; Hawkins et al., 2011). To our knowledge, the link between chemical types and their morphological differences have not been used as a basis to definitively differentiate populations of rooibos previously.

This present study thus aimed to determine the chemical fingerprints of fifteen previously uncharacterised populations via UPLC-MS. This was followed by a selection of ecotypes of unique or interesting metabolite content that were assayed for their antibacterial activity against *Staphylococcus aureus* and *S. epidermidis.* Because cultivated rooibos is often included in skin care products, antimicrobial activity of the four selected wild rooibos ecotypes was thus tested against these two commensal skin microbes (Claudel et al., 2019; Khorvash et al., 2012). As the phenolic content of rooibos is correlated to its antioxidant activity, it was hypothesised that populations with higher metabolic content would have stronger bioactivity in the various antioxidant assays performed, than those of lower metabolic content. This study has enabled us to highlight the most pharmacologically active ecotype/s in terms of antimicrobial activity and antioxidant potential using a metabolomics-driven approach.

## Materials and methods

### Plant material

**Fig. 1:**
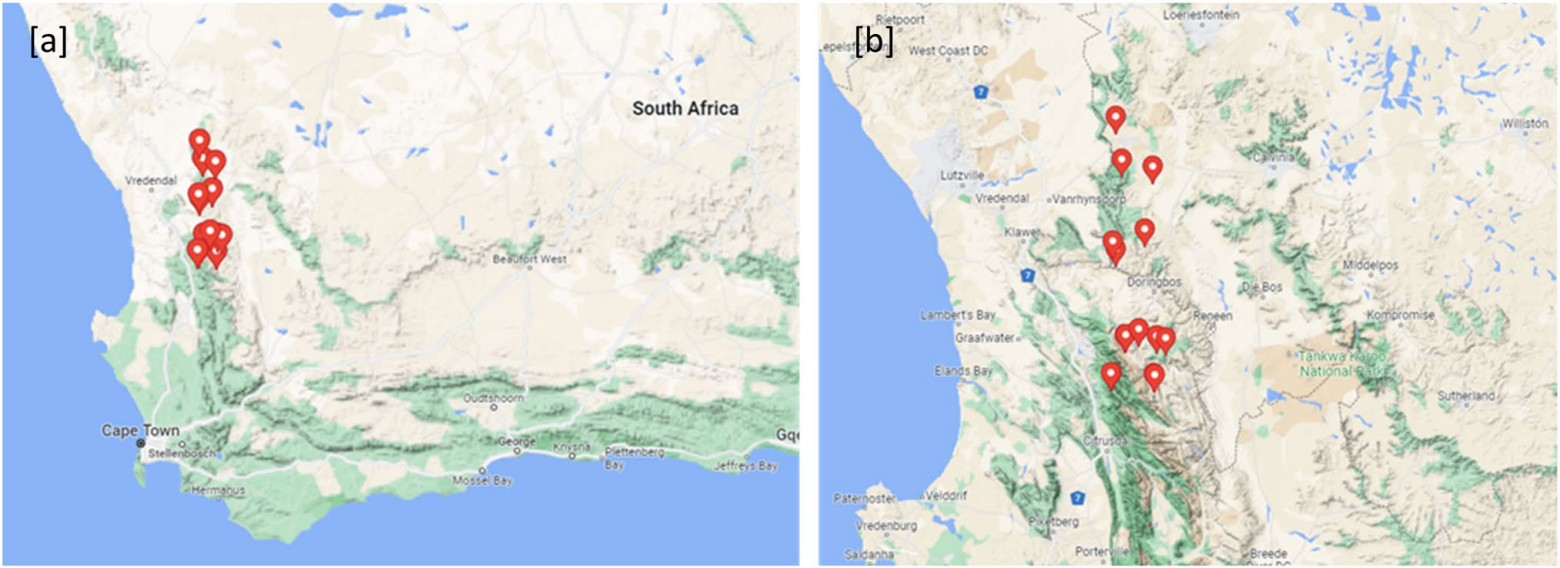
**a** Map of South Africa showing the locality of the 15 wild rooibos sampling sites in the Western Cape and Northern Cape; **b** Collection sites in different areas of Cederberg (more Southerly located pins) and Nieuwoudtville areas of South Africa (more Northerly located pins). The red pin drops indicate the exact location where the plant material was collected for this study

Collections of wild rooibos plants were gathered from four localities in Nieuwoudtville and the Suid Bokkeveld in the Northern Cape at various sites with permission from the Heiveld Cooperative and Indigo organisations (Fig. 1). Field harvests in the Northern Cape were conducted mid-February 2018 (February 10th to 19th). The exact locations of these wild ecotypes are known by the small-scale farmers in the areas. Field guides from local communities assisted with the identification of ecotypes of these plants. An additional two localities were donated by the Heiveld Cooperative, totalling six collection sites from the Northern Cape (Table 1). Accessions from the Western Cape were also collected in the Cederberg mountainous region in April 2021 (Fig. 1), with a flora collection permit issued by CapeNature (Permit no.: CN35-28–16,270). Nine populations were collected in the Cederberg region, two from the Heuningvlei area, two from Eselbank, close to Wupperthal, two from the mountain tops above the Biedouw Valley, one from within the Biedouw Valley and two from the Jamaka mountain area, close to Algeria. All ecotypes were of the wild variants which are known to possess morphological and physiological forms differing between the respective regions. The guidelines described in the Malgas et al. (2010) and Hawkins et al. (2011) were used as a reference (Table 1). The species collected was confirmed as *A. linearis* by Nokwanda Makunga (PhD) (botanist) and Rhoda Malgas (PhD) (a plant ecologist), in agreement with identifications performed by traditional knowledge holders and local farmers who cultivate rooibos. Referral to herbarium specimens that have been deposited at the Department of Botany and Zoology was also used to guide the confirmation of wild rooibos plants collected for this study. The Cederberg populations were sampled between 4 and 28 km apart, separated by the Cederberg mountains, while the Northern Cape populations were between 6 and 45 km apart. This was to ensure the representation of the entire wild rooibos distribution area (total distance of 100 km). From each site, 10–15 individual plants were used to represent a population and approximately 200 g of foliage was harvested. All collected plant material was left to air dry in brown paper bags at room temperature until further analysis and the first extraction experiments were conducted 3 months after the collection date. The work of Stander et al. (2017) and Stander et al. (2019) refers, as a chemotaxonomy fingerprinting approach (described below) was further used to confirm the integrity of the collected samples. Rooibos-specific chemical markers (aspalathin, nothofagin, isoorientin, and orientin, to name a few) were used to provide evidence that all the collected plant materials were indeed *A. linearis* ecotypes.

**Table 1:** Wild rooibos sampling sites and details of collections from the Cederberg region of the Western Cape and the Suid Bokkeveld of the Northern Cape

Sample site/region	Reason for collection	Growth form	GPS coordinates	Elevation (m)	Specimen Voucher
Blomfontein, Nieuwoudtville	Unusual/unique morphology and colouring	Prostrate	31° 32’S 19° 13’E	740	*A.lin*_B2018
Dobbelaarskop, Nieuwoudtville	Unusual/unique morphology and colouring	Prostrate	31° 47′ S 19° 11′ E	720	*A.lin*_D2018
Eselbank site 1, Cederberg (Wupperthal)	Previously untested	Prostrate	32° 21′ S 19° 13″ E	935	*A.lin*_E12021
Eselbank site 2, Cederberg (Wupperthal)	Previously untested	Prostrate	32° 21′ S 19° 13′ E	935	*A.lin*_E22021
Heuningvlei site 1, Cederberg	High quantities of compounds	Bush type	32° 12′ S 19° 05′ E	930	*A.lin*_H2018
Heuningvlei site 2, Cederberg	Previously uncharacterised	Upright/bush type	32° 12′ S 19° 05′ E	870	*A.lin*_H22021
Jamaka site 1, Cederberg (Algeria Valley)	Previously untested	Upright ‘Kriedouw’ type with blue-green leaves	32° 21′ S 19° 02′ E	390	*A.lin*_J2018
Jamaka site 2, Cederberg (Algeria Valley)	Unusual/unique morphology and colouring	Upright ‘Kriedouw’ type with blue-green leaves	32° 21′ S 19° 01′ E	400	*A.lin*_J22021
Landskloof, Nieuwoudtville	Unusual/unique morphology and colouring		31° 50’S 19° 02’E	390	*A.lin*_LK2018
Matarakopje, Nieuwoudtville	Unusual/unique morphology and colouring		31° 31’S 19° 04’E	760	*A.lin*_Ma2018
Melkraal, Nieuwoudtville	Unusual/unique morphology and colouring		31° 20’S 19° 03’E	810	*A.lin*_M2018
Pieke, Cederberg (Biedouw Valley mountain-top)	Previously untested	Prostrate	32° 12′ S 19° 16′ E	845	*A.lin*_P2021
Sonderwaterkraal, Nieuwoudtville	Unusual/unique morphology and colouring		31° 52′ S 19° 3′ E	370	*A.lin*_SWK2018
Wegbreek (Biedouw Valley)	Previously untested	Upright	32° 10′ S 19° 09′ E	450	*A.lin*_WB2021
Welbedacht, Cederberg (Biedouw Valley mountain-top)	Previously untested	Bush type	32° 12′ S 19° 14′ E	870	*A.lin*_W2021

Growth form categories after Hawkins et al. (2011)

### Metabolomics

**Fig. 2:**
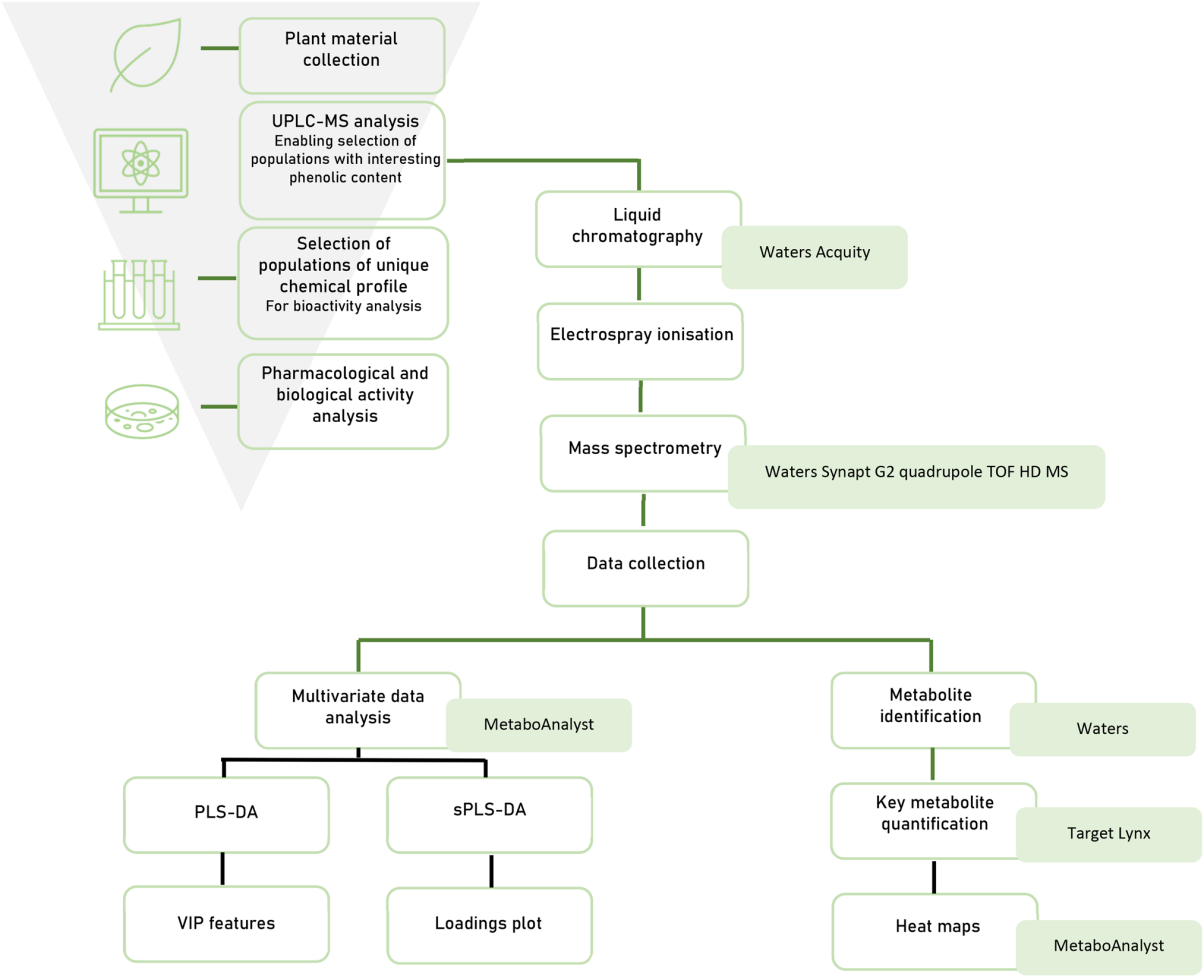
Research flow diagram explaining the methods from collection through metabolomic and bioactivity analyses, including the workflow of the semi-targeted metabolomics approach of this study on wild rooibos. The green lines indicate the steps that follow the process of analyses, starting from the top and moving downwards. The black lines indicate the methods that comprise the various types of analyses

A semi-targeted metabolomics approach was chosen, using a high-resolution ultra-performance liquid chromatography mass spectrometry (UPLC-MS) method as the analytical tool based on the method of Stander et al. (2017) to assess the phenolic profiles of the wild rooibos ecotypes. The metabolomics workflow is depicted as a flow diagram (Fig. 2).

### Phytochemical extractions for metabolomics

The extraction method of Stander et al. (2017) was followed. Briefly, dried plant material (6 g) from each individual was finely ground with a Retsch Mill (PM100 Plantary Ball Mill) and 2 g of dried ground powder was transferred to a 50 mL centrifuge tube. The ground tissue was then extracted with 15 mL of 50% (v/v) methanol in water containing 1% (v/v) formic acid overnight at room temperature. The extraction was continued with ultrasonication of the samples at room temperature for 1 h at 0.5 Hz using a sonicator (Bransonic 32 Ultrasonic Cleaner). The extracts were then centrifuged at 3000×*g* for 5 min to separate the supernatant from the plant material. All extractions were done in triplicate.

### High resolution UPLC-MS

High resolution Ultra-Performance Liquid Chromatography Mass Spectrometry (UPLC-MS) analysis was enabled by the Waters Synapt G2 Quadrupole time-of-flight (QTOF) mass spectrometer (MS) connected to a Waters Acquity Ultra-Performance Liquid Chromatograph (UPLC) (Waters, Milford, MA, USA) and samples were run at the Central Analytical Facility (CAF) at Stellenbosch University using previously reported methods (Stander et al., 2017, 2019). For this particular study, a negative mode electrospray ionisation was performed using the previously set parameters for rooibos samples by Stander et al. (2017), namely a cone voltage of 15 V, desolvation temperature of 275 °C and desolvation gas at 650 L/h. A Waters HSS T3, 2.1 × 100 mm, 1.8 µm column was used for metabolite separation. The mobile phase that was used consisted of solvent A: 0.1% (v/v) formic acid and solvent B: acetonitrile with 0.1% (v/v) formic acid. An injection volume of 2 µL was chosen. The flow rate was 0.3 mL/min with a column temperature at 55 °C. The instrument was calibrated with sodium formate. All analyses were performed in triplicate. Data was obtained in resolution mode by scanning from *m/z* 150 to 1500 in MS^E^ mode. Using MS^E^ mode, two channels were used to acquire data, where one was set at a low collision energy (4 V) and the second channel was used in order to obtain fragmentation data, using an energy ramp from 40 V-100 V.

### Multivariate data analyses and metabolite identification

Multivariate data analysis performed using the online MetaboAnalyst v4.0 application to determine the relationship between the localities and the metabolites using principal component analysis (PCA) to reduce the dimensionality of the data. The algorithms assist with peak picking, elimination of noise and retention time alignment. This model implemented pareto-scaling. Principal component analysis revealed the chemical individuality of the various wild rooibos populations. Scores plots were generated and those chemicals that were responsible for PCA clusters were included in the list of metabolites that were tentatively identified for each population.

### Tentative metabolite identification

The tentative identification of chosen metabolites was based on mass spectra, elemental composition, peak integration and metabolite databases (Metlin, Phenol-Explorer, PubChem), an in-house database and literature assisted with tentative chemical assignment. Some metabolites were regarded as unknown as no chemical identities could be putatively assigned to these chemicals. All chemical assignments were based on their accurate mass and retention time (e.g.: Unknown 369_6.68) and in the case of those chemicals that have been previously recorded, available literature and databases were used to confirm their putative identities. Key metabolites were identified from the total metabolites observed and a heatmap was generated using the online MetaboAnalyst v4.0 application. In this study, individual spectra attest to the complex nature of rooibos extracts with phenolics occurring between 0 and 28 min (RT: retention time).

### Metabolite quantification

The key metabolites were selected and quantified using the TargetLynx application manager (Waters MassLynx™) with the aim of comparing the relative concentrations of each compound to each of the other populations. These key compounds were chosen based on three criteria:

1. those identified from the loadings plot causing the separation of the clusters in the PCA;
2. novel/ newly identified compounds; and,
3. unknown metabolites.

Rutin was chosen as the reference metabolite where a standard curve was constructed (10–200 ppm) using 5 reference points. This enabled for the elucidation of relative concentrations of the key compounds using an external standard quantification method (ESTM). Relative abundance, associated with peak areas, generated from the TargetLynx application manager (Waters MassLynx™) was converted to concentration using the standard curve of rutin. These concentrations were used as comparison amongst different populations. Key metabolites were quantified to relative concentrations in mg/kg dry weight (wt).

### Univariate data analysis

An analysis of variance was employed by means of a nested one-way ANOVA using GraphPad Prism v8.2.0. In instances where the data did not follow a normal distribution, Kruskal Wallis analysis was employed as a post-hoc test to determine statistical differences amongst the different means. Otherwise, a Tukey post-hoc test was used to infer means for all normally distributed data.

### Phytochemical extraction for antioxidant and antimicrobial analyses

Three different populations from the Cederberg region were selected for further study based on their chemical fingerprints as these populations had higher relative concentrations of metabolite and these included Welbedacht, Eselbank site 1 and Eselbank site 2 populations. Additionally, the Jamaka population was selected for its unique morphology and colouring and none of the Northern Cape populations were selected for the bioactivity assays. For each extraction, the dried material (10 g) was ground to a fine powder with the use of liquid nitrogen and a pestle and mortar. Following this, 50 mL of 1:1 dichloromethane (DCM): methanol (v/v) solvent was added to the plant material and left for 72 h under constant agitation on a shaker (Wirsam Scientific, Cape Town) at 125 rpm. The maceration was filtered with a Buchner funnel and No. 541 Whatman filter paper and air-dried under a fume hood. Extracts were stored at 4 °C until use.

### Total phenolic content and total flavonoid content

The total phenolic content (TPC) and total flavonoid content (TFC) of the four populations was assessed, as well as the antioxidant activity via three assays. The phenolic content of the extracts was determined via the Folín-Ciocalteu (Folin-C) method, as outlined in Magangana et al. (2021) and Fawole et al. (2012). Sample extracts were tested in triplicate. A gallic acid ethanolic solution (0–0.014 mg/mL) was used to generate a standard curve based on which results were quantified and thereafter expressed in mg gallic acid equivalents (GAE) per g dry mass.

The methods described by Magangana et al. (2021) and Yang et al. (2009) were used to obtain the flavonoid content. Briefly, sample extracts were mixed with 50% (v/v) methanol, vortexed and subjected to ultrasonication, followed by centrifugation at 4000×*g*. A mixture of extract solution, distilled water, and 5% (w/v) sodium nitrite was left to sit for 6 min, after which 10% (w/v) aluminium chloride and 1 mM sodium hydroxide were added. Distilled water was added to bring the mixture to 3 mL. Absorbance of each sample was measured at 510 nm with the use of a UV–visible spectrophotometer (Thermo Scientific Technologies, Madison, WI, USA). Sample extracts were tested in triplicate and results expressed in mg catechin equivalents per g extract as based on a 0–0.5 µg/mL catechin calibration curve.

### Antioxidant activity

The 2,2-azino-bis (3-ethylbenzothiazoline-6-sulphonic acid) (ABTS^+^) assay was used to measure antioxidant activity via radical cation decolourisation performed with the following brief method described in Chirinos et al. (2013). A potassium persulphate solution (2.6 mM) was prepared and added to a 7.4 mM ABTS solution (1:1, v/v), followed by incubation in a dark environment at 25 °C for 12 h. The resulting ABTS^+^ solution was diluted with methanol until an absorbance measurement of 0.70 ± 0.02 at 750 nm was obtained. Mixtures of ABTS^+^ and samples were allowed to react for 6 min, after which absorbances were measured at 750 nm with the use of a UV–visible spectrophotometer (Thermo Scientific Technologies, Madison, WI, USA) and results presented in Trolox equivalents per g sample (µmol Trolox/g dry matter). Samples were tested in triplicate.

The ferric ion reducing antioxidant power (FRAP) assay was performed according to Magangana et al. (2021) to assess the ability of the extracts to reduce a ferric-tripyridyltriazine complex to the ferrous form, thereby eliciting a colour change. A freshly prepared FRAP solution [300 mM sodium acetate buffer (pH 3.6), 10 mM 2,4,6-tri(2-pyridyl)-s-triazine (TPTZ) solution and 20 mM ferric chloride solution] was thoroughly mixed and incubated at room temperature for 30 min under dark conditions. An 80% (v/v) methanol solution was used as a negative control and a 10 µg/mL Trolox solution as a positive control. Samples were dissolved in 50% (v/v) methanol and added to the FRAP solution to be incubated in the dark at 25° C for 30 min. Absorbances were measured at 593 nm with the use of a UV–visible spectrophotometer (Thermo Scientific Technologies, Madison, WI, USA) and results presented as µmol Trolox/g dry matter.

The free radical-scavenging abilities were tested by a 2,2-Diphenyl-1-picryl Hydrazyl (DPPH) assay according to Karioti et al. (2004). Sample extracts were mixed with methanol and a 0.1 mM DPPH solution and incubated in the dark at room temperature for 30 min. Samples were tested in triplicate and compared to an ascorbic acid positive control (1000 µg/mL) and a 50% (v/v) methanol negative control. Absorbances were measured at 517 nm with the use of a UV–visible spectrophotometer (Thermo Scientific Technologies, Madison, WI, USA) and quantified based on a 0–1500 µM standard curve; results are presented as µmol Trolox/g dry matter.

All results are presented as mean values (± SD) of three independent triplicate experiments. ANOVA was performed using GraphPad Prism 4.0 software. Tukey post-hoc testing was used to determine significant differences in results (significance level of p < 0.05).

#### Antimicrobial activity

Antimicrobial activity of the selected extracts (Welbedacht, Jamaka, Eselbank site 1 and Eselbank site 2) was determined against *S. aureus* (ATCC 12600) and *S. epidermidis* (ATCC 35984) according to the broth dilution method outlined in Mphahlele et al. (2016) and Lall et al. (2013), with slight modifications to determine the minimum inhibitory concentration (MIC) of the extracts. Bacterial cultures were grown for 24 h in Mueller Hinton (MH) broth (Sigma-Aldrich, Missouri, USA) and diluted in MH broth to a spectrophotometer reading of OD_600_ = 0.08 AU (1.5 × 10^8^ CFU/ml) or McFarland turbidity. The McFarland-adjusted *S. aureus* was further diluted in MH broth at a ratio of 1:500 (3.0 × 10^5^ CFU/ml) due to its rapid growth rate. Streptomycin (Sigma-Aldrich, Missouri, USA) was used as the positive control at a concentration range of 0.39 to 25 µg/mL. Extract stock solutions were added to the wells and serially diluted to obtain a concentration range of 39.06 to 2500 µg/mL. Bacterial suspension and 200 µg/mL 2, 3, 5-triphenyltetrazolium chloride (Sigma-Aldrich, Missouri, USA) visibility reagent were added and the plates were incubated at 37 °C for 16–18 h, after which visual determination of MIC values was performed, where a clear colour indicates no bacterial growth and a pink colour indicates the presence of bacterial growth. The MH broth was used as a positive control and 2.5% v/v DMSO was used as a vehicle control. A MIC value of 100 µg/mL and less was considered potent activity (de Canha et al., 2020) and data is presented as mean values ± SD of three independent triplicate experiments.

## Results

### Metabolomics

**Fig. 3:**
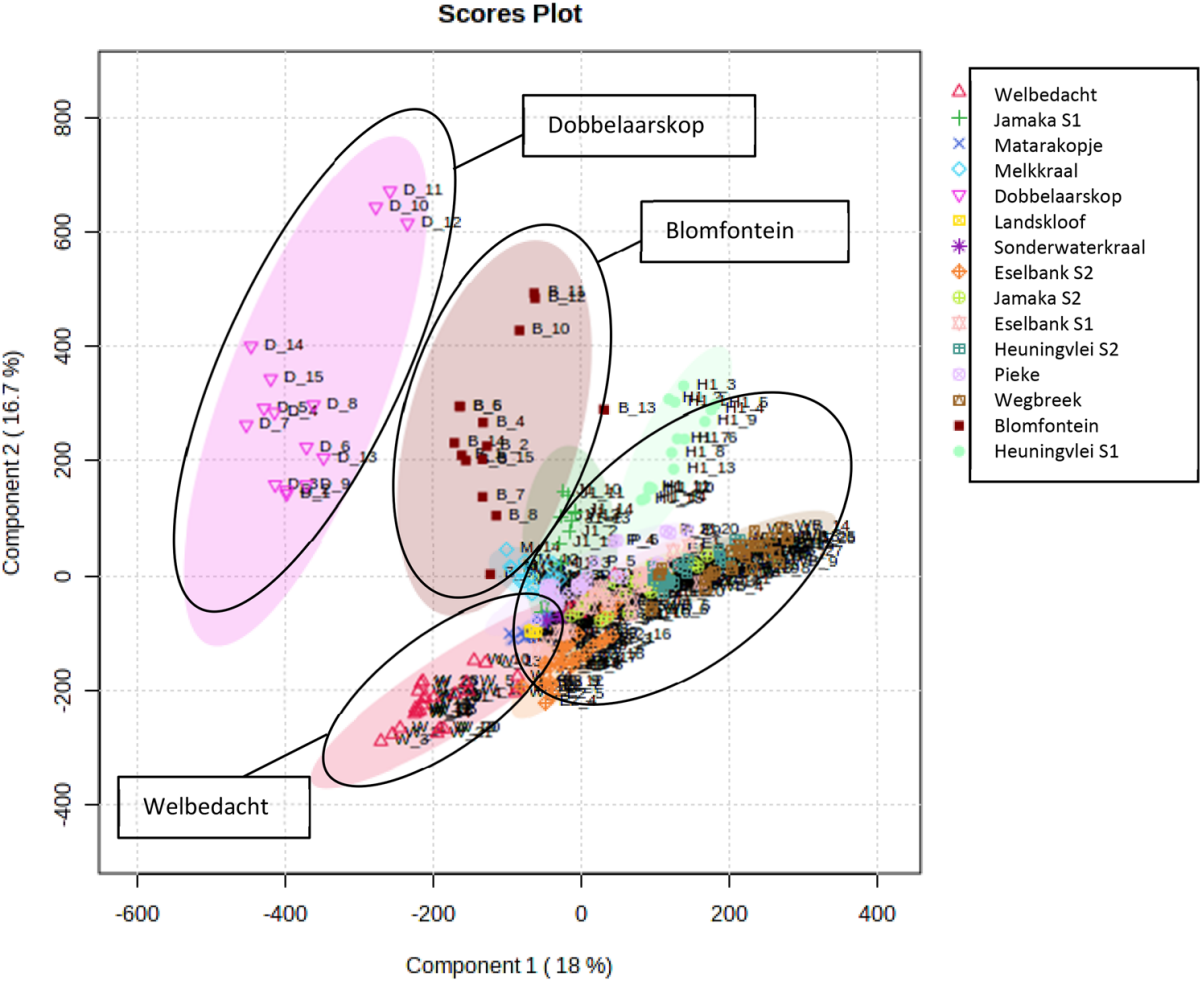
PLS-DA 2D scores plot of all 15 populations with four main clusters (I, II, III, IV) demarcated

A total of 55 major metabolites were tentatively identified in the fifteen wild rooibos populations that were sampled. These major metabolites were used to differentiate between the different chemotypes studied and provide further confirmation of the identification of the collected plant material as *A. linearis* [Fig. 3 and Supplementary data (Table A)]. Although many of the identified chemicals were present in all the rooibos samples, quantities varied considerably with respect to the biomarker compounds in different samples. Three populations, Dobbelaarskop, Blomfontein and Welbedacht, displayed significant variation in metabolite content in comparison to the other populations (Fig. 3). These populations had notably higher relative amounts of metabolites, however the more prevalent metabolites are not the same for all three of these populations, thus further emphasising the chemotypic variation displayed among rooibos ecotypes.

**Fig. 4:**
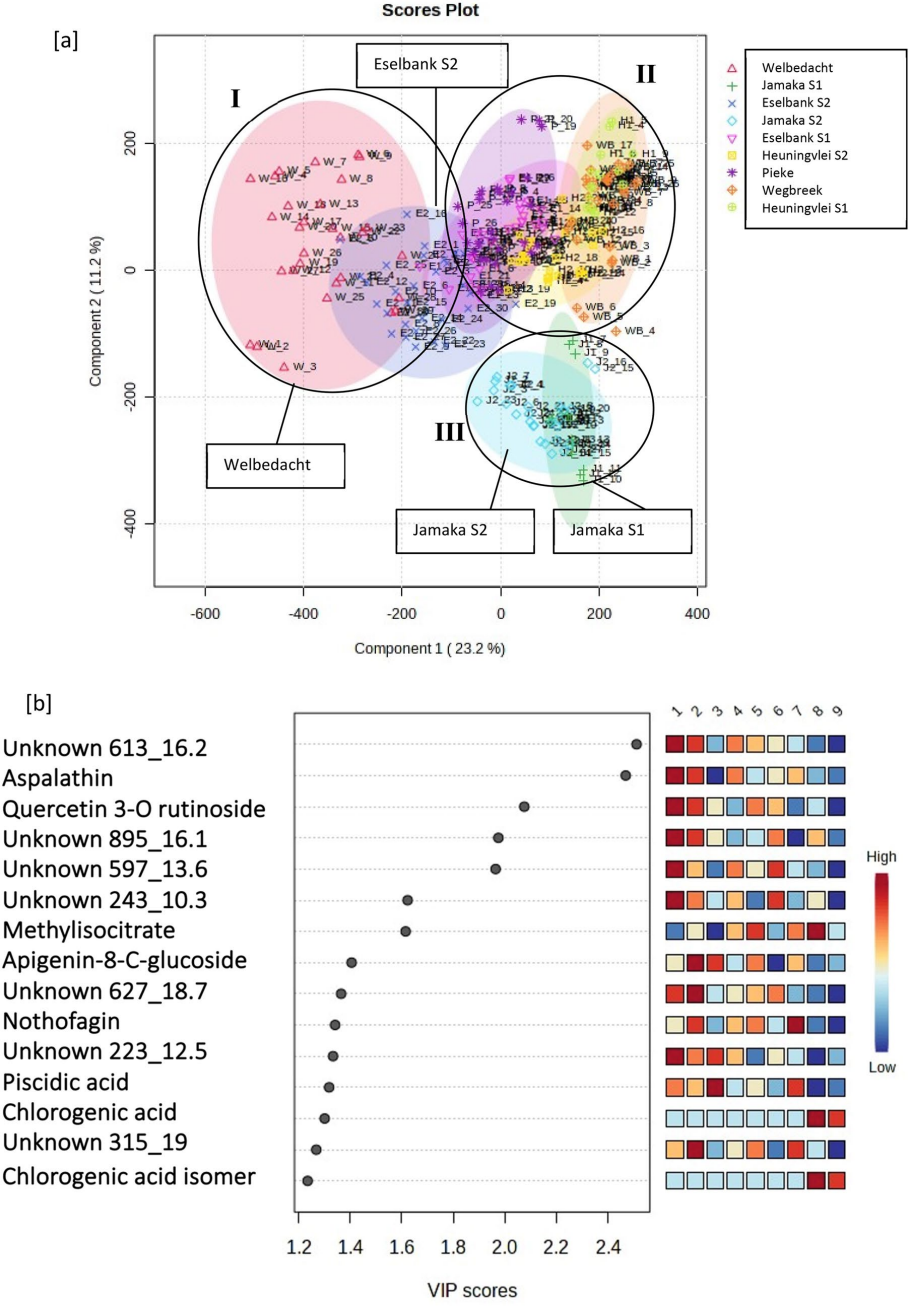
**A** PLS-DA 2D scores plot of the Cederberg populations depicting three main clusters (I, II, III); **b** PLS-DA variable-importance-in-projection (VIP) features (numbers 1 to 9 correspond to the following populations respectively: Welbedacht, Eselbank S2, Jamaka S2, Eselbank S1, Heuningvlei S2, Pieke, Wegbreek, Heuningvlei S1, Jamaka S1)

The PLS-DA 2D scores plot of the Cederberg populations formed three main clusters (Fig. 4). Cluster I was made up primarily of the Welbedacht population and part of Eselbank site 2. Cluster II included Pieke, Heuningvlei sites 1 and 2, Eselbank site 1 and part of Eselbank site 2, and Wegbreek. Cluster II was made up of Jamaka sites 1 and 2. There is a relatively high amount of overlap of all the populations except Welbedacht and the Jamaka sites.

**Fig. 5:**
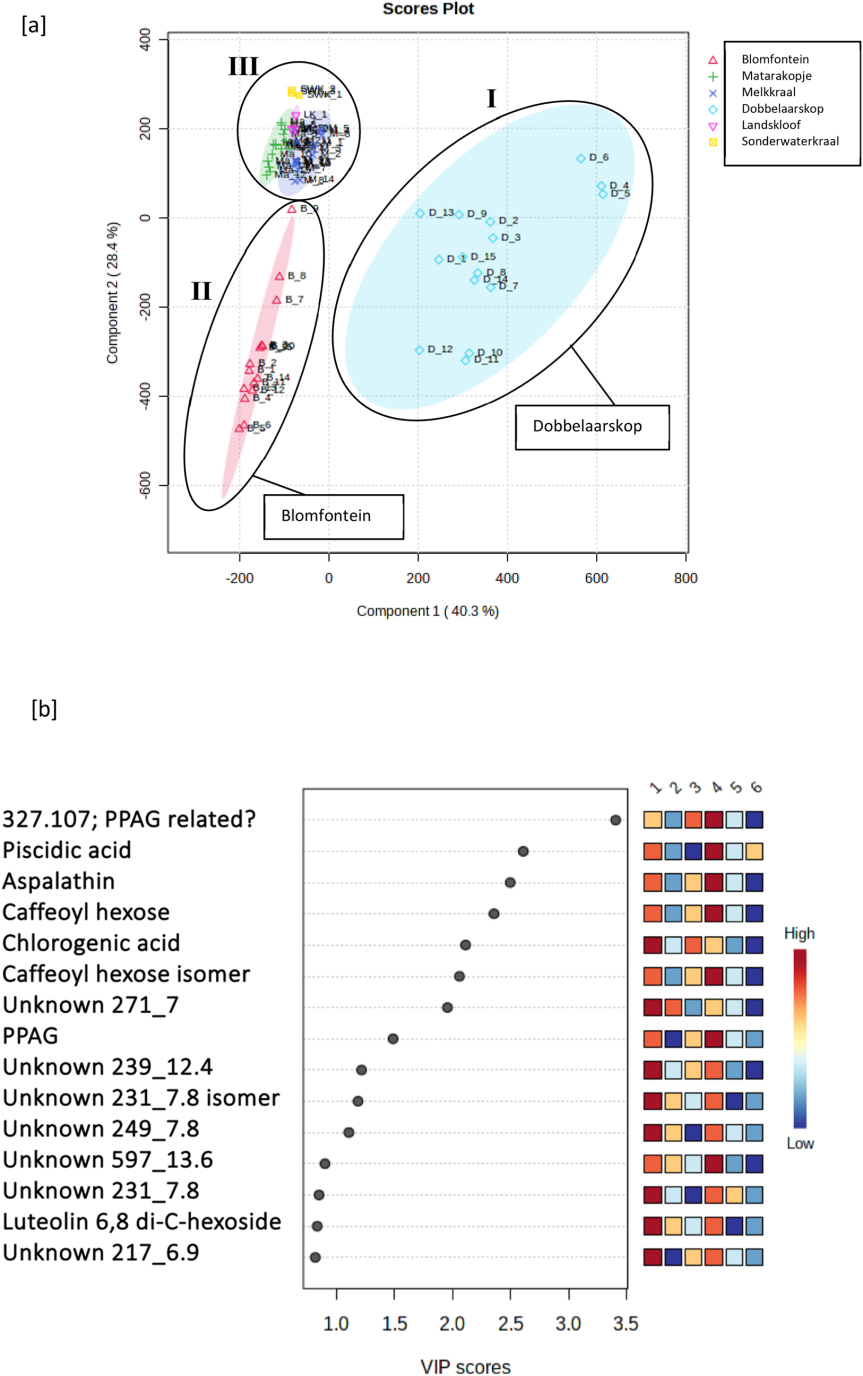
**a** PLS-DA 2D scores plot of the Northern Cape populations with three main clusters (I, II, III) depicted; **b** PLS-DA variable-importance-in-projection (VIP) features (numbers 1 to 6 correspond to the following populations respectively: Blomfontein, Matarakopje, Melkkraal, Dobbelaarskop, Landskloof, Sonderwaterkraal)

Three main clusters, I, II and III are visible. Figure 5 shows the clear interpopulation variance between Dobbelaarskop (I) and the other five Northern Cape populations (II and III). Also visible is the intrapopulation variance in the Dobbelaarskop, depicted by the larger size of this cluster, which could potentially be separated into two or three smaller subclusters. The Blomfontein population (II) showed relatively high variance along PC2 but lower along PC1. The other four populations (Matarakopje, Melkkraal, Landskloof and Sonderwaterkraal) displayed low interpopulation variance depicted by the closely clustered pattern (III). Several phenolic acids, such as piscidic acid, fukuiic acid and citric acid as well as a flavonoid glucoside were important for grouping the Cluster III plants (Matarakopje, Landskloof and Sonderwaterkraal) together.

**Fig. 6:**
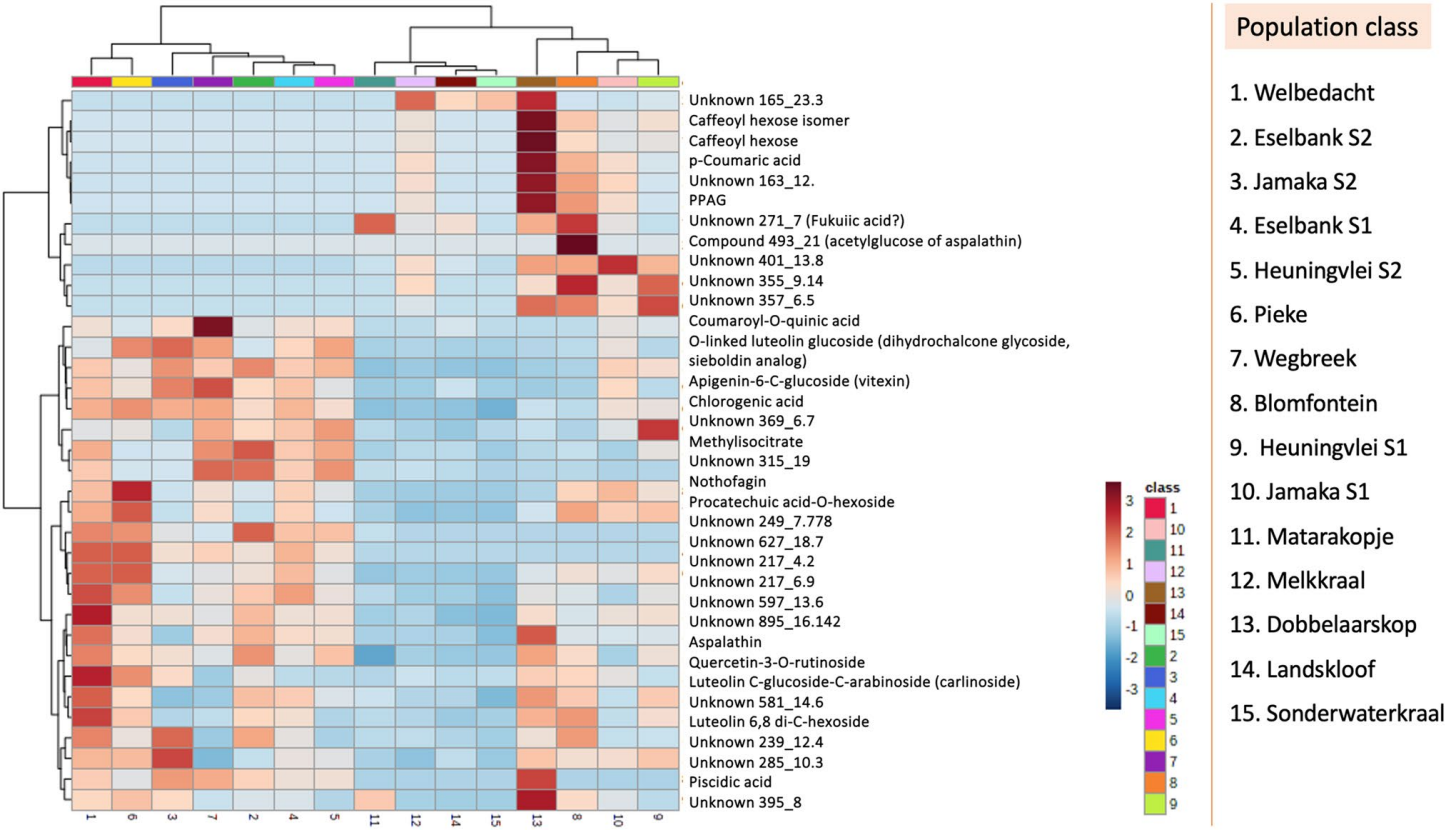
Heatmap of metabolites in all 15 populations of *A. linearis*

Blomfontein and Dobbelaarskop appear to be more closely related in terms of metabolite content to the Cederberg populations than other Northern Cape populations (Fig. 6). This appears to correlate with the geographical location of these specific Northern Cape populations, which are located further south than most of the other Northern Cape populations (i.e.: closer to the Cederberg region). The Landskloof and Sonderwaterkraal are located further south than the Dobbelaarskop and Blomfontein populations, however, these populations only have one sample in triplicate whereas the other populations had more samples. Thus, the integrity of these results may be compromised to a degree.

The two Jamaka populations show a high degree of similarity, despite their collection dates being three years apart. The same is not seen for the Heuningvlei populations, which were also collected three years apart. The two Eselbank populations were collected at the same time on the sa me mountaintop but about 1–2 km apart and so the variation seen here is expected.

Although many of the identified chemicals were present in all the rooibos samples, quantities varied considerably in terms of the chemical markers that are widely used in industry. Those metabolites that had consistently low concentrations were collected from Jamaka; however, this population was unique with regards to its morphology and appearance, in particular the blue-green colour of the leaves and was thus included in the populations selected for bioactivity analysis. Other populations selected included the Welbedacht and two Eselbank sites which all displayed relatively high amounts of several key metabolites (Fig. 6). For example, the Welbedacht population had high relative ion intensities for several quercetin derivatives and unknown phytochemicals. Of the group of plants examined in this study, the Heuningvlei, Jamaka, Welbedacht, Pieke, and Wegbreek populations were monitored for the first time, and their phytochemical profiles are novel contributions to knowledge on the species.

### Polyphenolic content

Analysis of the total phenolic and total flavonoid content of the extracts showed that the highest content of both TPC and TFC was found in the Welbedacht population (Table 2), at 36.938 ± 0.20 mg GAE/g dry matter and 20.395 ± 0.30 mg CE/g dry matter, respectively. The lowest content of both phenolics and flavonoids at 23.791 ± 0.20 mg GAE/g dry matter and 7.0168 ± 1.01 mg CE/g dry matter, respectively, were both found in the Eselbank site 1 population. Significant (p < 0.05) differences were noted between the phenolic contents of the populations as well as between the flavonoid contents. The higher TPC and TFC values recorded in the Welbedacht population corroborate the findings of the UPLC-MS analysis which indicated a relatively large number of key metabolites present in higher concentrations in this population than in the other populations tested (Fig. 6).

**Table 2:** Total phenolic and total flavonoid content of the four selected populations

Population site	TPC (mg GAE/100 g dry weight)	TFC (mg CE/100 g dry weight)
Eselbank site 1	23.791 ± 0.19	7.0168 ± 1.01
Eselbank site 2	33.816 ± 0.47	17.799 ± 0.26
Jamaka site 2	28.578 ± 0.21	14.222 ± 0.20
Welbedacht	36.938 ± 0.20	20.395 ± 0.30

Values are means ± SD of triplicate (n = 3) determinations

### Antioxidant activity

The antioxidant activity of the four populations (Eselbank sites 1 and 2, Jamaka and Welbedacht) was analysed via three different methods, each measuring a different mechanism involved in antioxidant capacity. The ABTS and DPPH assays measure the radical scavenging ability, while the FRAP assay measures the reducing power of the extracts in the reduction of a Fe^3+^-TPTZ complex to a coloured Fe^2+^-TPTZ complex. Higher absorbances indicate higher activity in all three cases. These results obtained were then compared with the TPC and TFC of each population to find potential correlations between the datasets.

**Fig. 7:**
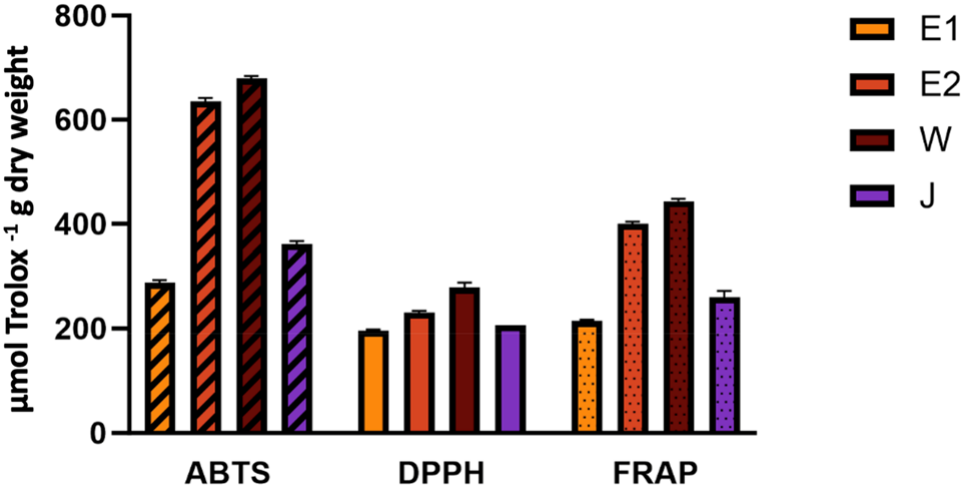
Antioxidant activity of the four selected populations of wild *A. linearis* as measured by the ABTS, DPPH, and FRAP antioxidant assays

Significant differences in the radical scavenging ability and reducing power of the extracts, as measured by all three assays (ABTS, FRAP, and DPPH) were revealed for the samples tested in this study [Fig. 7, (*p* < 0.05)]. The highest activity for all three assays was exhibited by the Welbedacht population, at 679.894 ± 3.43 µmol Trolox/g dry matter, 443.834 ± 3.43 µmol Trolox/g dry matter, and 278.861 ± 7.85 µmol Trolox/g dry matter, for the ABTS, FRAP, and DPPH assays, respectively.

For all the antioxidant assays and the TPC and TFC analyses, Eselbank site 2 had the second highest values after Welbedacht, followed by Jamaka site 2 and, finally, Eselbank site 1. High correlation was found between both the TPC and TFC with the antioxidant assay results. Correlation values of TPC with ABTS, FRAP and DPPH were 0.97, 0.98 and 0.91 respectively, while the correlation values of TFC with ABTS, FRAP and DPPH were 0.91, 0.93 and 0.85, respectively. These values indicate significantly high levels of correlation between both the phenolic and flavonoid content and the antioxidant activity.

### Antimicrobial activity

Dichloromethane: methanol (1:1) extracts of the four selected populations yielded average to low antimicrobial activity against *S. epidermidis* and *S. aureus* (Table B; Supplementary materials). The Welbedacht and Jamaka site 2 extracts yielded visually observed minimum inhibitory concentration (MIC) values of 625 µg/mL against *S. epidermidis*, which were slightly lower than those observed for the Eselbank sites (i.e.: slightly higher activity of the former). The lowest MIC (i.e.: strongest activity) against *S. aureus* was recorded for the Welbedacht extract, at a value of 312.5 µg/mL. The observed MIC values showed comparatively low activity in relation to the antibiotic control, streptomycin (MIC of 1.563 µg/mL). Consistency of results was observed across all replicates. Visual observation of the media and bacterial controls indicated zero contamination.

## Discussion

### The phytochemical profile of rooibos

The chemical profile of rooibos is known to be complex and mainly composed of phenolic acids and flavonoids (Stander et al., 2017). There are a few of these chemicals in rooibos that are unique to *Aspalathus linearis* such as aspalathin and phenylpropenoic acid glucoside (PPAG) (Diallo et al., 2015; Stander et al., 2017). This study has tentatively identified several new chemicals that have never been described in rooibos before as well as those which are yet to be characterised (i.e.: those compounds labelled in the format ‘Unknown X_Y’) [Fig. 6 and Supplementary data (Table A)]. This highlights the importance of a comprehensive analysis of these poorly characterised wild populations, as differences in chemical profiles correlate to potential in pharmacological activity and relate back to ecological factors in the natural environment.

New chemotypes of rooibos create an opportunity for future bioprospecting activities of this species (Stander et al., 2017). Local communities within the study area have often claimed the superiority of certain ecotypes of rooibos, in having greater health benefits and being attributed with higher resilience to pests, pathogens and drought than their cultivated counterparts (Malgas et al., 2010).

The multivariate analyses clearly illustrate phenolic variation of the wild rooibos populations (Fig. 6). Several metabolites were identified with the same mass but differed in retention time such as E-PPAG and PPAG (Table 2). It was thus imperative to correctly identify all major metabolites using accurate mass, spectral data (m/z) and metabolite databases. The 55 metabolites identified represent both commonly found metabolites, such as citric acid and coumaric acid, and those which are unique to rooibos, as well as some unknown metabolites.

The Northern Cape populations were characterised by plants that were larger in size (approximately 2 m in height) and of a more salignus type morphology with longer leaves and a sparser crown. In contrast, the plants growing in the Cederberg region appeared to be smaller bushes (30 cm high) with slightly shorter leaves. The Jamaka type was even seen to have grey-green coloured foliage, which was not seen in any other populations sampled. *Aspalathus linearis* displays phenotypic plasticity amongst the various populations. Thus, differentiating between ecotypes can be complex. Within the Cederberg, the populations differed quite substantially, as seen in the taller, almost tree-like, Wegbreek population, compared to the low-lying Eselbank populations. Both qualitative and quantitative differences in the chemical constituents of rooibos based on the phenolic fingerprints can be applied to differentiate rooibos ecotypes that are currently not commercialised. Some of the significant differentiating chemical signatures in the wild ecotypes include fukuiic acid, uralenneoside, citric acid, piscidic acid, methylisocitrate, and three as yet unidentified compounds; unknown 493_21.1, unknown 395_9.08 and unknown 597_13.63 (Fig. 6).

Rooibos extracts are highly complex in their phytochemical constituents. As a result, the specialised metabolites with the bioactivity in rooibos are not necessarily all known but rooibos is rich in specialised metabolites that belong to the phenolic class. Several of the phenolics found in rooibos are known to occur at higher concentrations in unfermented rooibos and this green rooibos extracts have been shown to exhibit strong antioxidant activity (Breiter et al., 2011).

Some of the metabolites that were important in differentiating between ecotypes include PPAG, caffeoyl hexose, piscidic acid, and chlorogenic acid that have been shown previously to occur in rooibos extracts (Fig. 6). However there are several other phytochemicals that have not been characterised before and these are important to purify and identify in the future as they may also impart unique and/or specific bioactivities, thus making it important to know which rooibos ecotypes should be recommended for their pharmacological value. More importantly, it would be useful to have the structural identities of the unknown compounds that are present at higher concentrations in those populations with stronger bioactivity and determine whether this bioactivity is correlated to the presence of these unknowns (Fig. 6).

Secondary metabolite profiles in plants respond to environmental changes showing the power of phenotypic plasticity of plants as an adaptive strategy to changing microclimatic conditions (Kronholm & Collins, 2016; Malgas et al., 2010). Factors that are known to influence plant metabolite biosynthesis include temperature, soil nutrients, microbial diversity/relationships, circadian rhythm, water availability, and plant-animal interactions such as (e.g., interactions with herbivores, and pollinators), to mention a few (Bundy et al., 2009). The quality of cultivated rooibos is affected by different production seasons and this was described in detail in the work of Joubert et al. (2012) and several key chemical markers including aspalathin, orientin, vitexin, ferulic acid, quercetin-3-*O*-robinobioside and rutin, to mention a few, were assayed in samples that were collected over a three year period. No significantly obvious changes to the total phenolic content were noted. The growth patterns of wild rooibos, similarly to cultivated rooibos, are highly influenced by different seasons and wild plants are better able to tolerate summer conditions that are characterised by elevated temperatures with long periods of water being unavailable for the plants (Lötter & le Maitre, 2014).

Unique metabolite profiles resolved for each of the wild populations are directly influenced by the varying environmental factors of each location. Soil composition, rainfall and elevation are key factors that may influence the metabolites produced by the different populations. The soil at the Dobbelaarskop location is thought to have higher clay content compared to the other localities, as the farm is located to the eastern edge of the Suid Bokkeveld, approaching the Tankwa Karoo (Smith et al., 2018). Soils at Landskloof and Sonderwaterkraal are deep, sandy, porous soils that look like beach sand. While the sites may differ with regard to soil types, the one thing that might be common amongst these locations is rainfall. There is a dramatic drop in elevation over the 80–100 km between the escarpment at Nieuwoudtville, and the farms in the south in the elbow of the Kobee and Doring rivers. The interesting thing about this site is that it is located in the south-western corner of the Suid Bokkeveld. It is one of the more arid sites, partly because of its location near the edge of the Suid Bokkeveld where elevation and rainfall are relatively much lower than at Nieuwoudtville on the escarpment up north, but also because it edges into the arid Tankwa Karoo (Malgas et al., 2010). The site at Heuningvlei, further south, sits at higher elevation and experiences higher rainfall. Ecologically, one might have expected stronger concentrations of metabolites for Dobbelaarskop, given the harsh growing conditions there, and the opposite for the Cederberg populations. It is interesting to note that, despite their clustering with Blomfontein and Melkkraal (Fig. 5), the three sites located at the southernmost part of the Suid Bokkeveld, are about 30 km and 60 km away from those populations, respectively.

Fynbos species are adapted to fire disturbance, with an average fire return interval of 12 years for the western interior of the CFR where study sites were located (Kraaij & Van Wilgen, 2014). As with other Fynbos species, *A. linearis* ecotypes have evolved fire survival strategies such as sprouting and seeding, that are mutually exclusive in rooibos (Malgas et al., 2010; Marais et al., 2014). These strategies largely influence the growth and development of the plant and highlight how fire plays an important role in the morphology and life history of these fynbos endemics. Fire adaptive strategy, or life history, may be a significant explanatory factor in the interpretation of results for this study. It is known that chemical profiles are correlated to the morphological, phenological and biochemical characteristics of *A. linearis* (Brooks et al., 2021; Potts et al., 2017; van Heerden et al., 2003; Van der Bank et al., 1999). This study extends that correlation to congruencies between life history (reseeder or resprouter) and metabolomic pathways. It is quite useful to understand that chemical profiles are also correlated to the phenological strategy used by *A. linearis* in terms of its ecological survival and that developmental stage and plant age play a key role in metabolite biosynthesis (Petrussa et al., 2013). As this data shows, there is also a clear separation between resprouters and reseeders with respect to their metabolite profiles. Those plants that are defined as resprouters are mostly found in more arid areas, and in more porous, acidic soils. Similar distribution patterns have been reported for other Fynbos plants with congeneric reseeding and resprouting. In rooibos, this translates into ecologically distinct ecotypes that are also genetically distinct, and that are typically exclusively found within a population/locality, (i.e., geographic isolation resulting in genetic barriers) (Brooks et al., 2021; Hawkins et al., 2011; van der Bank et al., 1995). These localities also vary in environmental conditions such as soil profile, temperature and average rainfall which may also influence metabolite production. Recently, Brooks et al. (2021) confirmed the occurrence of distinct wild rooibos haplotypes using a chloroplastic marker. These were linked to biogeographic location and life history strategies as reseeders were genetically differentiated from resprouters that were chosen for that particular study. The authors further indicated that isolation by distance may influence gene flow as wild rooibos populations are small, have a patchy distribution plus they exhibit low-to-moderate intrapopulation genetic diversity.

The Cederberg climate can be described as hot semi-arid according to the Kӧppen Geiger climate classification system, with an average annual temperature and rainfall of 19.4 °C and 224 mm, respectively. Using the same system, the Nieuwoudtville area can be described as cold semi-arid, with an average temperature and rainfall of 16.2 °C and 304 mm, respectively (CSIR, 2015). The Dobbelaarskop population is slightly separated from the other Northern Cape populations in the Suid Bokkeveld by a gorge, acting as a geographical barrier (Malgas et al., 2010). This separation, together with the different soil type of the Dobbelaarskop site could explain some of the significant metabolomic differences. It is also important to note that some of the metabolites were significantly different in the Heuningvlei population. Again, some of these differences could be explained by environmental influence. The Cederberg populations are not vastly different from the Northern Cape populations, except for the Dobbelaarskop and Blomfontein populations, in their metabolomic content, as seen in the PLS-DA plot (Fig. 3). The ecological type of that area is typically single-stemmed and faster growing as opposed to multi-stemmed, slower growth like that of the Dobbelaarskop population. The Northern Cape populations are also most often sprouting types, whereas the Cederberg commonly have reseeding types, though resprouting types are present. The interpopulation variation between clusters I and II, and III and IV respectively (Fig. 3), which shows no distinct separation between the Northern Cape and Cederberg populations, may be due to the presence of both reseeding and resprouting types in both regions. The Welbedacht population of the Cederberg has relatively high variation in metabolic content in comparison to the other populations. The slight overlap in metabolites between the Welbedacht and Pieke populations can most likely be attributed to the closeness of the site localities, however, from the heatmap (Fig. 6) the Welbedacht population has a relatively higher number of key metabolites present at higher concentrations than most of the other populations sampled.

Metabolomic studies are often used in the identification and quantification analysis of chemical markers of food and medicinal plants (Aszyk et al., 2018). At this stage, the rooibos industry is primarily interested in aspalathin and nothofagin as these compounds are used to monitor the quality of plant material. Heuningvlei, Matarakopje and Melkkraal showed significantly higher concentrations of citric acid in comparison with the other populations. Overall, the Cederberg populations had higher concentrations of all metabolites than the Northern Cape populations. This may be due to the nature of the soils in the southern regions of the rooibos distribution area, particularly the soil pH levels. The pH levels are higher in the sandstone-derived soils of the northern rooibos production areas, especially in the southern parts of the Northern Cape, while the soil pH levels in the southern Cederberg regions is expected to be higher than the Northern Cape (Chimpango et al., 2013). Citric acid is thus an important distinguishing chemical feature of the Cederberg group and may become a useful biomarker for quality assurance in rooibos. The recent work of Stander et al. (2017) confirmed the presence of PPAG in 18 rooibos populations. The present study corroborates this data and includes a quantitative analysis of PPAG, adding a new dimension to differentiate rooibos types based on their biogeography. It appears to be a precursor for the biosynthesis of many flavonoids (Muller et al., 2018). Phenylalanine is important as a precursor compound for PPAG as it leads to the production of phenylpropanoic acid. Phenylpropenoic acid is then enzymatically converted to hydroxypropanoic acid, directly leading to the accumulation of PPAG. PPAG occurs at low levels in unfermented rooibos, yet hot water extractions and infusions result in higher levels. More recently, PPAG has demonstrated antidiabetic activity, yet further studies are needed (Muller et al., 2018).

Several chemicals have not previously been reported in rooibos and genetic-to-molecular networks that control their biosynthesis in rooibos have not yet been characterised. It is only recently that efforts to provide a reference genome of rooibos have been made (Brooks et al., 2021; Stander et al., 2019). Many phenolic metabolites resolved in rooibos populations in this study are well-known as being strong antioxidants such as luteolin and its derivatives, quercetin and its isomers (Bramati et al., 2003; Santos et al., 2016).

Following the metabolomics analysis, four populations of interest were chosen for inclusion in pharmacological studies. The Welbedacht, Jamaka site 2, Eselbank site 1 and Eselbank site 2 populations were selected on the basis of their unique metabolomic content and/or unusual morphology (in the case of the Jamaka population). This was assessed based on the PLS-DA plot and VIP features identified by this analysis. Due to accessibility reasons, only the Cederberg populations were included in this selection process.

### The pharmacological potential of rooibos

Of the four selected populations, the highest TPC and TFC was found in the Welbedacht population. This is consistent with the findings of the UPLC-MS analysis, in which the highest quantities of metabolites were also found to be present in the Welbedacht population. A high TPC value generally correlates with a high antioxidant capacity, as seen in previous studies, as phenols are of the most common naturally occurring antioxidants (Sharadanand Phatak et al., 2014; Tan & Lim, 2015). Results obtained from the DPPH, FRAP and ABTS assays all displayed high correlation with the TPC and TFC of the samples. Potent antioxidant activity by a variety of mechanisms (e.g., metal chelation, ROS quenching, free radical scavenging) is linked to flavonoid content. Flavonoids are often linked to a variety of pharmacological activities, such as antimicrobial, anticancer, anti-inflammatory and antioxidant (Magangana et al., 2021; Zonyane et al., 2020). These compounds are able to scavenge free radicals which could otherwise lead to the onset and progression of various health conditions and diseases such as cancers. It is known that plants commonly produce antioxidants, however, the degree of antioxidant activity may be higher when species-specific metabolites with specialised antioxidant activity are present (Zonyane et al., 2020). The antioxidant activity of natural antioxidant substances is uncertain and condition dependent, therefore it is important to perform both in vitro and in vivo analysis before making claims with regards to potentially therapeutic antioxidant substances (Gupta et al., 2020; Qi et al., 2022). In this particular study, an in vitro assay was thus conducted. There is a known correlation between antioxidant activity (as determined by ABTS scavenging) and aspalathin content. Pure aspalathin has pro-oxidant activity which has a close correlation with the flavonoid and dihydrochalcone content of rooibos extracts (R^2^ = 0.977 and 0.971) (Marnewick, 2009). Radical scavenging is made possible by the presence of hydroxyl residues in metabolites such as ellagitannins, a type of polyphenol, which enable quenching of radical species. The significance of the high antioxidant activity of the populations is related to the prevention and treatment of cancer incidence and progression, both of which are made worse by the presence of ROS, which act as key signalling molecules in these processes. Flavonoids have been shown to have transcription suppression and signal transduction modulation of cyclooxygenase 2, the overexpression of which is commonly associated with cancers (Slika et al., 2022).

All the samples tested showed antioxidant activity as measured by all three assays, and different strengths of activity were recorded for all four populations. Extracts were diluted when high absorbance readings were obtained, to achieve clear and accurate measurements within the necessary parameters. The Welbedacht sample exhibited the highest antioxidant activity in all three assays. The DPPH, ABTS, and FRAP assays all proceed via the single electron transfer (SET) mechanism (Tan & Lim, 2015). This explains the consistency in the results obtained across the populations for all three assays. The similarity of these results can be used to ensure accuracy and confirm that the SET method of antioxidant action is used by the samples tested. The DPPH and ABTS assays record the free radical scavenging activity of the antioxidant whilst the FRAP assay measures the reducing capacity of the antioxidant on the oxidant (ferric ions) via a redox reaction (Sharadanand Phatak et al., 2014; Tan & Lim, 2015). Asalathin and nothofagin are known strong antioxidants that occur most prominently in unfermented (green) rooibos. Breiter et al. (2011) tested an aspalathin-enriched fraction and green unfermented rooibos in a human clinical trial. This extract was shown to be bioavailable in the gastrointestinal tract and an increase in the antioxidant effects in human subjects were associated with its consumption.

*Staphylococcus epidermidis* and *S. aureus* are two common elements of the human skin microbiome. *Staphylococcus aureus* colonises the skin as an asymptomatic pathogen, while *S. epidermidis* will on occasion promote infection, despite its mutualistic nature, and in other instances, has been found to aid in wound-healing, eliciting a positive effect on the immune barrier responses and thereby further benefiting other cutaneous systems (Chen et al., 2018; Krutmann, 2009). *Staphylococcus epidermidis* may on occasion produce molecules which are able to selectively inhibit the growth of *S. aureus*, raising the question of potential antagonism between the two species (Chen & Tsao, 2013).

Due to the behavioural aspects of the bacterial species tested (pathogenic/commensal), the extracts from the populations overall inhibited the growth of *S. epidermidis* but had slightly higher activity against *S. aureus*. The Welbedacht and Jamaka populations exhibited the highest activity against *S. epidermidis* (625 µg/mL), while the Welbedacht population had the highest activity against *S. aureus* (312.5 µg/mL). This provides evidence of the superior antibacterial activity of the Welbedacht population compared to the other wild populations that have been tested in this study. However, this activity is still relatively low to moderate, as an MIC value of 100 µg/mL and less is generally accepted as indicative of a strong antibacterial activity (De Canha et al., 2020). The overall low antimicrobial activity could be viewed as a favourable attribute, as the use of rooibos in skin products will have little effect on the skin microbiome, thereby facilitating a healthy skin environment.

The metabolites present in wild rooibos are likely responses to environmental factors. The evolution of phenolic metabolites in plants is still not well understood but may recognise the importance of phenolics and their protective mechanisms. Many of these metabolites are known for their powerful antioxidant properties and were therefore worthy of further investigation and characterisation. Potent antioxidant activity by a variety of mechanisms (e.g.: metal chelation, ROS quenching, free radical scavenging) is linked to flavonoid content. High levels of correlation between both the phenolic and flavonoid content and the antioxidant activity of the Welbedacht, Jamaka, and Eselbank site 1 and 2 and populations have been shown. There is a known correlation between antioxidant activity (as determined by ABTS scavenging) and aspalathin content. Pure aspalathin has pro-oxidant activity which has a close correlation with the flavonoid and dihydrochalcone content of rooibos extracts (R2 = 0.977 and 0.971) (Marnewick, 2009). Radical scavenging is made possible by the presence of hydroxyl residues in metabolites such as polyphenols, which enable quenching of radical species.

The wild rooibos populations of this study displayed no distinct chemical profiles between the resprouter populations of the Northern Cape versus the reseeder populations of the Cederberg, however, the Dobbelaarskop and Welbedacht populations of the Northern Cape and Cederberg respectively, showed definite interpopulation variation. Overall, these two populations appeared to have higher concentrations of the majority of the metabolites compared to the other populations, while the populations that had consistently lower concentrations were collected from Matarakopje, Sonderwaterkraal, Melkkraal and Landskloof (i.e., all from the Northern Cape) (Figs. 3, 5). Overall, the plants collected from the Cederberg region had higher metabolite content than the Northern Cape populations. Currently, very little is known regarding the Dobbelaarskop and Welbedacht populations of plants as there is minimal background information with respect to plants found at Dobbelaarskop (Chimphango et al., 2016), and none for Welbedacht, making this a first-time analysis of this and some of the other populations. Some of the significant differentiating chemical signatures across all populations include fukuiic acid, uralenneoside, citric acid, piscidic acid, and two as yet unidentified compounds; unknown 493_21.11 and unknown 597_13.63.

Market development of wild rooibos ecotypes cannot be decoupled from sustainable harvesting practices, as reflected in the current status of certain wild rooibos populations as many of these face unpredictable impacts of climate change, urban expansion and agricultural developments in the areas where these populations grow, possibly threatening their future existence as valued biocultural assets(Joubert et al., 2017; Wynberg, 2017).

## Conclusion

This metabolomics study has enabled us to obtain a fingerprint of the plant metabolites present in the various populations of *A. linearis* where prior phytochemical information was not available and thus four of these from the Cederberg area were chosen for downstream bioactivity analyses. The resprouter populations of the Northern Cape seem to be of the same chemical lineage versus the reseeder populations of the Cederberg, where greater instances of phytochemical differentiation were apparent. Overall, the plants collected from the Cederberg region had higher metabolite content than the Northern Cape populations. Currently, very little is known regarding the Dobbelaarskop and Welbedacht populations of plants as there is no background information with respect to plants found at these sites, making this a first-time analysis of these and some of the other populations. The significant antioxidant activity linked to the metabolomic content of the wild rooibos populations such as the Welbedacht collection, could render these populations as useful ingredients in a number of herbal formulations/remedies. Isolation and structural characterisation of the unknown metabolites in rooibos ecotypes could broaden current phytochemical information regarding rooibos population extracts.

The overall effect of the wild population extracts on the skin appears to be highly promising, as seen by the high antioxidant activity and minimal disruption to the skin microbiome. In particular, the extract 49 from the Welbedacht population appears to be superior in its activity to the other populations in most assays tested in this study. This information contributes to indigenous knowledge and boosts the commercial potential of this South African endemic species. A comparison of the wild types with the cultivated type would be valuable to confirm the potential need for cultivation and conservation efforts of the wild types to be initiated, should they be superior in bioactivity to the currently cultivated type. All of these above-mentioned studies, alongside the present study, together deepen our understanding of the biology and ecology of this commercially and culturally valued species. This research offers a novel contribution of linking the phytochemistry with the morphology and bioactivity of wild rooibos ecotypes. Lastly, the chemical diversity that is attached to different populations of *A. linearis* in the wild corroborates indigenous knowledge of the rural communities that benefit from a livelihood linked to this plant and the claims that the wild populations exhibit useful ethnobotanical properties.

### Supplementary Information

Below is the link to the electronic supplementary material.

Supplementary file1 (DOCX 149 kb)
