# Supplementary material for: Metabolomic profiling of wild rooibos (Aspalathus linearis) ecotypes and their antioxidant-derived phytopharmaceutical potential
Source: Metabolomics. 2024 Apr 14;20(3):45. doi: 10.1007/s11306-024-02103-4 (PMC11016507; doi:10.1007/s11306-024-02103-4)
Supplement: Supplementary file 1 — Supplementary file1 (DOCX 149 kb) [file 11306_2024_2103_MOESM1_ESM.docx]

**Supplementary materials**


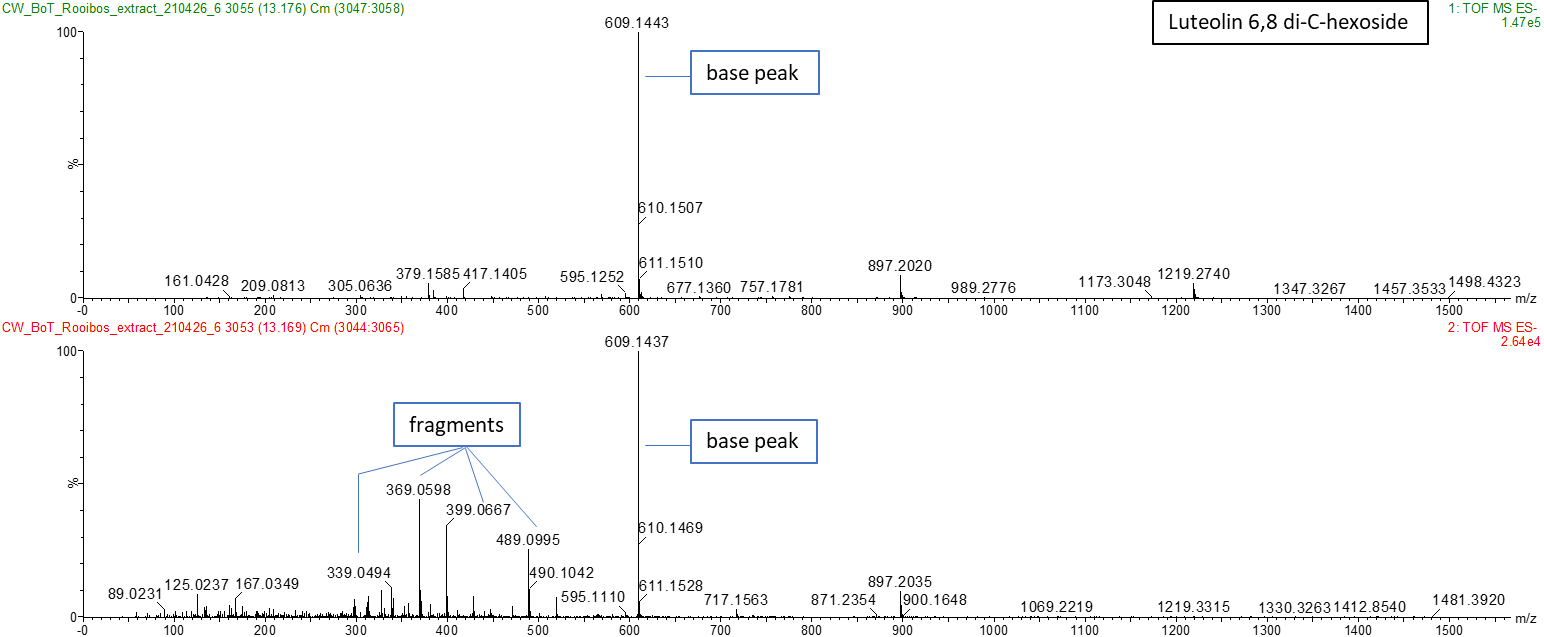


Fig S1 Separation of phenolic compounds in *A. linearis* extracts. MS^E^ spectra showing fragmentation pattern of luteolin 6,8 di-C-hexoside of *m/z* 609, at the retention time of 13.192


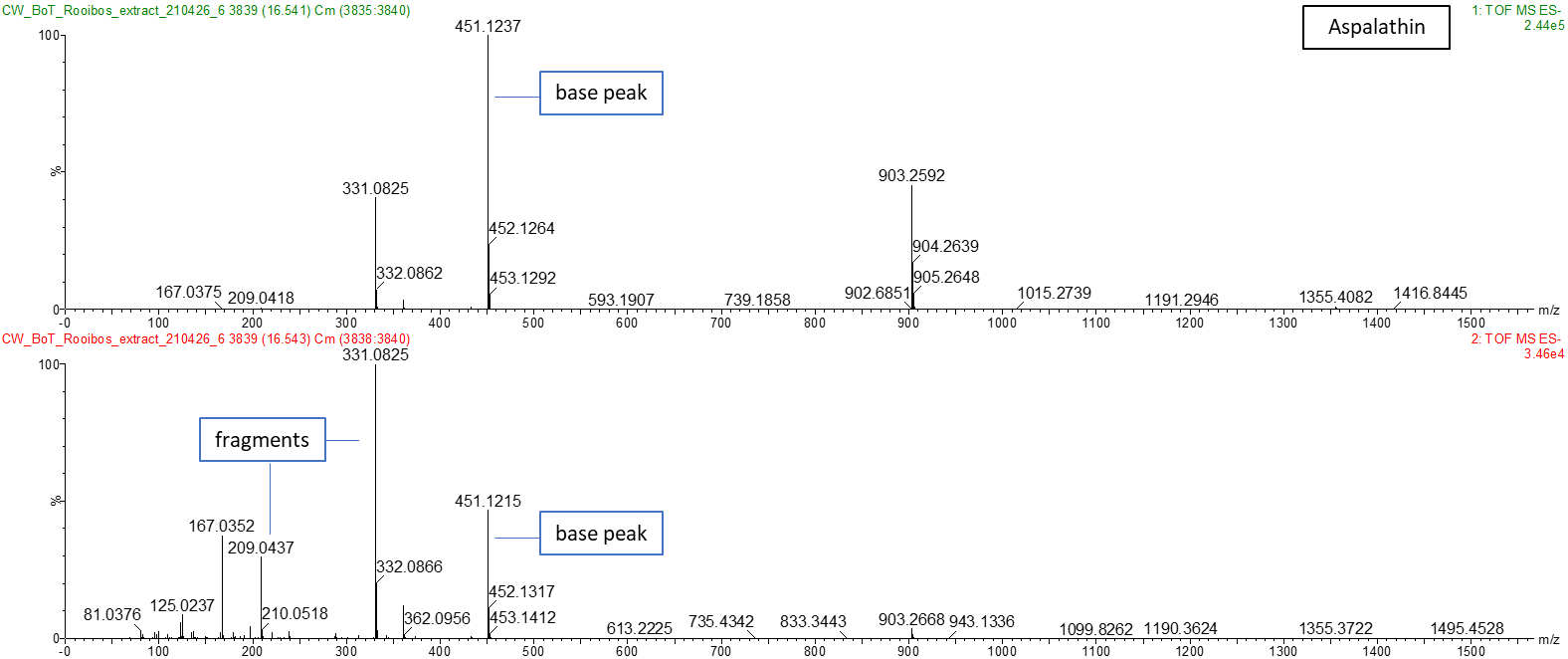


Fig S2 Separation of phenolic compounds in *A. linearis* extracts. MS^E^ spectra showing fragmentation pattern of aspalathin of *m/z* 451, at the retention time of 16.54


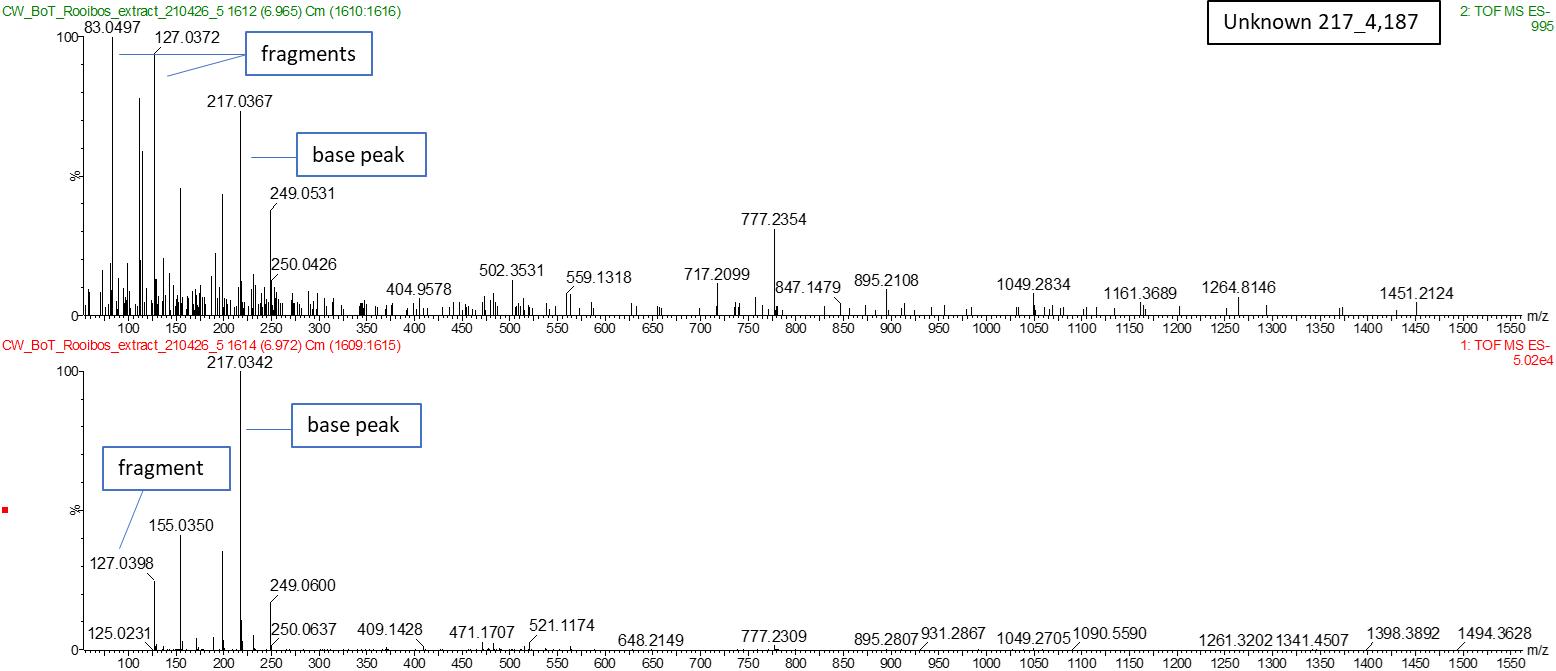


Fig S3 Separation of phenolic compounds in *A. linearis* extracts. MS^E^ spectra showing fragmentation pattern of Unknown 217_4.187 of *m/z* 217, at the retention time of 4.187

**Table A** Phenolic metabolites tentatively identified in wild rooibos populations using LC-MS metabolite profiling

| Phenolic compounds identified in wild rooibos populations | | | | | |
| --- | --- | --- | --- | --- | --- |
| Mass m/z | Compound | RT (min) | Chemical formula [M-H]^-^ | MS/MS fragments | References |
| 191.0191 | Citric acid | 3.26 | C_6_H_7_O_7_ | 87, 111 | Stander et al., 2017 |
| 217.0349 | Unknown 217_4.187 | 4.187 | C_12_H_9_O_4_ | 127, 111, 83 |  |
| 357.0813 | Unknown 357_6.5 | 6.5 | C_15_H_17_0_10_ | 151, 195, 313 | Stander et al., 2017 |
| 243.0516 | Unknown 243_6.639 | 6.639 | C_10_H_11_O_7_ | 152, 185, 181 |  |
| 369.1397 | Unknown 369_6.68 | 6.68 | C_14_H_25_O_11_ | 243, 237, 161, 265 |  |
| 217.0342 | Unknown 217_6.962 | 6.962 | C_8_H_9_O_7_ | 83, 127 |  |
| 271.0446 | Unknown 271_7 (Fukuiic acid?) | 7.02 | C_11_H_11_O_8_ | 181, 123, 109 | Stander et al., 2017 |
| 341.0862 | Caffeoyl hexose | 7.73 | C_15_H_17_0_9_ | 135, 179 | Stander et al., 2017 |
| 249.0613 | Unknown 249_7.778 | 7.778 | C_6_H_17_O_10_ | 185, 127 |  |
| 155.0344 | Unknown 155_7.889 | 7.889 | C_7_H_7_O_4_ | 127 |  |
| 383.1553 | Unknown 383_8.019 | 8.019 | C_15_H_27_O_11_ | 161, 155, 127, 199, 315 |  |
| 315.0708 | Procatechuic acid-O-hexoside | 8.086 | C_13_H_15_O_9_ | 231, 153, 185, 181 |  |
| 255.0498 | Piscidic acid | 8.41 | C_11_H_11_0_7_ | 165, 179, 193 | Stander et al., 2017 |
| 395.1550 | Unknown 395_9.08 | 9.08 | C_16_H_27_O_11_ | 161 |  |
| 355.0658 | Unknown 355_9.14 | 9.14 | C_22_H_11_O_5_ | 153 | Stander et al., 2017 |
| 353.0870 | Chlorogenic acid | 9.84 | C_16_H_17_0_9_ | 191, 179 | Stander et al., 2017 |
| 205.0709 | Unknown 205_9.9 | 9.91 | C_8_H_13_0_6_ | 153, 158, 199 | Stander et al., 2017 |
| 285.0607 | Unknown 285_10.31 | 10.31 | C_12_H_13_O_8_ | 137, 243/152, 153, 197 |  |
| 299.0767 | Unknown 299_10.964 | 10.964 | C_13_H_15_O_8_ | 137 |  |
| 137.0242 | p-hydroxybenzoic acid | 10.964 | C_7_H_5_O_3_ | 125 | Stander et al., 2017 |
| 341.0862 | Caffeoyl hexose isomer | 10.98 | C_15_H_17_0_9_ | 135, 179 | Stander et al., 2017 |
| 337.0945 | Coumaroyl-O-quinic acid | 11.366 | C_16_H_17_O_8_ | 163, 119, 175, 115 |  |
| 463.0895 | Quercetin 3-galactoside | 11.443 | C_12_H_19_O_12_ | 175, 163, 125, 327 | Iswaldi et al., 2011 |
| 163.0395 | Unknown 163_12.015 | 12.02 | C_9_H_7_O_3_ | 125, 135, 161 |  |
| 325.0917 | PPAG | 12.27 | C_15_H_17_O_8_ | 163, 119, 91 | Stander et al., 2017 |
| 239.0554 | Unknown 239_12.4 | 12.4 | C_11_H_11_O_6_ | 125, 179, 167, 209, 195, 165, 191 |  |
| 609.1437 | Luteolin 6,8 di-C-hexoside | 13.192 | C_27_H_29_O_16_ | 369, 399, 489, 339 | Stander et al., 2017 |
| 597.1461 | Unknown 597_13.592 (Quercetin 3-O-[beta-D-xylosyl-(1- > 2)-beta-D-glucoside?) | 13.59 | C_26_H_29_O_16_ | 172, 153, 199 | Malongane et al., 2018. |
| 387.1653 | Unknown 387_13.85 | 13.85 | C_18_H_27_O_9_ | 205 |  |
| 449.1084 | (S)-eriodictyol-8-C-b-D-glucopyranoside | 14.031 | C_21_H_21_O_11_ | 193, 329, 135 | Stander et al., 2017 |
| 173.0450 | Unknown 173_14.142 | 14.142 | C_7_H_9_O_5_ | 135, 125, 163, 167, 149 |  |
| 449.109 | (R)-eriodictyol-8-C-b-D-glucopyranoside | 14.17 | C_21_H_21_O_11_ | 285, 329, 331, 359 | Stander et al., 2017 |
| 593.1497 | Apigenin-6,8-di-C-glycoside (vicenin-2) | 14.302 | C_27_H_29_O_15_ | 579, 353, 369, 447, 297, 161 | Stander et al., 2017 |
| 581.1506 | Unknown 581_14.579 | 14.579 | C_26_H_29_O_15_ | 329, 193, 135, 249, 371 |  |
| 579.1320 | Luteolin C-glucoside-C-arabinoside (carlinoside) | 15.058 | C_26_H_27_O_15_ | 369, 397, 275, 193, 489 | Stander et al., 2017 |
| 163.0387 | ρ-Coumaric acid | 15.24 | C_9_H_7_0_3_ | 119, 91 | Stander et al., 2017 |
| 443.120 | Unknown 443_15.552 | 15.552 | C_19_H_23_O_12_ | 267, 134, 193, 249, 331 |  |
| 325.092 | E – PPAG | 15.61 | C_15_H_17_O_8_ | 119, 161 | Stander et al., 2017 |
| 447.0899 | Luteolin6-C-glucoside (isorientin) | 15.932 | C_12_H_19_O_11_ | 327, 357 | Stander et al., 2017 |
| 895.1979 | Unknown 895_16.142 | 16.142 | C­_42_H_39_O_22_ | 447, 327 |  |
| 451.1228 | Aspalathin | 16.54 | C_21_H_23_0_11_ | 331, 361, 209 | Stander et al., 2017 |
| 449.1084 | Aspalalinin | 16.882 | C_21_H_21_O_11_ | 331, 167, 209, 125 | Stander et al., 2017 |
| 431.0995 | Apigenin-8-C-glucoside (vitexin) | 17.431 | C_21_H_19_O_10_ | 311, 300, 331, 283 | Stander et al., 2017 |
| 609.1455 | Quercetin 3-O rutinoside | 17.498 | C_27_H_29_O_16_ | 300, 271, 331 | Stander et al., 2017 |
| 431.0978 | Apigenin-6-C-glucoside (isovitexin) | 17.775 | C_21_H_19_O_10_ | 311, 300, 331, 283 | Stander et al., 2017 |
| 597.1819 | Catechin 3-O-rutinoside/ Phloretin-3’-5’-di-C-B-glucoside | 18.035 | C_27_H_33_O_15_ | 300, 463, 357, 271, 167, 387 | Stander et al, 2019. |
| 583.1604 | Unknown 583_18.543 | 18.543 | C_33_H_27_O_10_ | 167, 125, 331, 387, 417, 209, 193 |  |
| 627.1924 | Unknown 627_18.69 | 18.69 | C_28_H_35_O_16_ | 373 | Stander et al., 2017 |
| 451.1237 | O-linked luteolin glucoside (dihydrochalcone glycoside, sieboldin analog) | 18.916 | C_21_H_23_O_11_ | 167, 125, 209 | Stander et al., 2017 |
| 315.0869 | Unknown 315_19.032 | 19.032 | C_17_H_15_O_6_ | 167, 125, 209 |  |
| 435.1289 | Nothofagin | 19.14 | C_21_H_23_O_10_ | 315, 345 | Stander et al., 2017 |
| 493.1339 | Compound 493_21 (acetylglucose of aspalathin) | 21.11 | C_23_H_25_O_12_ | 331, 239, 167, 273 | Stander et al., 2017 |
| 491.1190 | Unknown 491_22.028 | 22.028 | C_23_H_23_O_12_ | 331, 193, 223, 167, 134, 209 |  |
| 725.3021 | Unknown 725_22.997 | 22.997 | C_35_H_49_O_16_ | 223, 193, 164, 149, 331 |  |
| 695.2915 | Unknown 695_23.302 | 23.302 | C_345_H_47_O_15_ | 193, 134, 223, 175 |  |

**Table B** Minimum inhibitory concentration values (µg/mL) of four *A. linearis* populations against *S. epidermidis* and *S. aureus*

| Population site | *S. epidermidis* (µg/mL) | *S. aureus* (µg/mL) |
| --- | --- | --- |
| Eselbank site 1 | 1250 | 1250 |
| Eselbank site 2 | 1250 | 1250 |
| Jamaka site 2 | 625 | 625 |
| Welbedacht | 625 | 312.5 |
